# Feasibility of a Stool State Check App Using AI During Bowel Preparation Before Colonoscopy: Multicenter Prospective Study (SCAN Study)

**DOI:** 10.2196/91274

**Published:** 2026-09-01

**Authors:** Atsushi Inaba, Kensuke Shinmura, Shozo Osera, Takahiro Yamada, Hiroaki Kon, Maki Kanazawa, Naoki Sugimura, Yasushi Sano, Hiroko Hosaka, Toshio Uraoka, Daiki Sato, Yusuke Yoda, Hiroyuki Takamaru, Yutaka Saito, Toshihiko Gocho, Atsushi Katagiri, Kazuhisa Yamaguchi, Takahisa Matsuda, Atsuki Imai, Hitomi Fujimoto, Hiroki Matsuzaki, Nobuyoshi Takeshita, Masashi Wakabayashi, Hiroaki Ikematsu, Tomonori Yano

**Affiliations:** 1 Department of Gastroenterology and Endoscopy, National Cancer Center Hospital East, 6-5-1 Kashiwanoha, Kashiwa, Chiba, 277-8577, Japan, 81 471331111, 81 471346865; 2 Department of Gastroenterology, Saku Central Hospital Advanced Care Center, Saku, Nagano, Japan; 3 Kon Internal Medicine Clinic, Chiba, Japan; 4 Gastrointestinal Center, Sano Hospital, Kobe, Hyogo, Japan; 5 Department of Gastroenterology and Hepatology, Gunma University Graduate School of Medicine, Maebashi, Gunma, Japan; 6 Department of Endoscopy, Saitama Cancer Center, Ina, Saitama, Japan; 7 Endoscopy Division, National Cancer Center Hospital, Tokyo, Japan; 8 Division of Gastroenterology, Department of Medicine, Showa Medical University School of Medicine, Tokyo, Japan; 9 Department of Internal Medicine Division of Gastroenterology and Hepatology, Omori Medical Center Toho University, Tokyo, Japan; 10 Department of Gastrointestinal Medicine, Aizawa Hospital, Matsumoto, Nagano, Japan; 11 Jmees, Inc., Kashiwa, Chiba, Japan; 12 Biostatistics Division, Center for Research Administration and Support, National Cancer Center, Tokyo, Japan; 13 Department of Gastroenterology, IMSUT Hospital, Institute of Medical Science, The University of Tokyo, Tokyo, Japan; 14 Medical Device Innovation Center, National Cancer Center Hospital East, Kashiwa, Japan

**Keywords:** artificial intelligence, bowel preparation, colonoscopy, mobile apps, smartphone, feasibility studies, workload, medical staff, surveys and questionnaires

## Abstract

**Background:**

Optimal bowel preparation (BP) is crucial for a successful colonoscopy. Although multiple factors influence BP quality, including patient adherence to laxatives and dietary instructions, the stool state during BP should be properly evaluated to perform a colonoscopy of sufficient quality. Therefore, we developed a smartphone app to evaluate a patient’s stool state during BP and a viewer to enable real-time monitoring by medical staff.

**Objective:**

This study aimed to assess the feasibility of performing colonoscopies of appropriate quality using the app-and-viewer system.

**Methods:**

This prospective observational study was conducted between November 2022 and December 2023, involving patients scheduled for colonoscopy at 10 Japanese institutions, comprising 6 tertiary hospitals, 3 regional general hospitals, and 1 community-based clinic. Patients who (1) underwent a colonoscopy at participating institutions, (2) were aged between 20 and 70 years, and (3) owned smartphones compatible with Android or iOS were included in the study. The patients downloaded the app on their smartphones and captured images of their stools during BP, while the medical staff reviewed the evaluation of the stools by the app via the viewer system. The primary end point was defined as the proportion of patients with a Boston Bowel Preparation Scale (BBPS) score of ≥6 among those who successfully used the app. Secondary end points included mean BBPS score, rate of an excellent BBPS score (≥8), adenoma detection rate, cecal intubation rate, and withdrawal time in negative colonoscopy. Additionally, we evaluated the usability of the app, medical staff workload burden with the app, and viewer usage via questionnaire surveys.

**Results:**

A total of 343 patients were enrolled, and 326 were ultimately included in the analysis. Overall, 99.1% (323/326, 95% CI 97.3%-99.8%) of the patients achieved the primary end point. The mean BBPS score was 8.5 (SD 1.0), and the proportion of excellent BBPS scores was 87.4% (285/326). The adenoma detection rate, cecal intubation rate, and mean withdrawal time in negative colonoscopy were 46.9% (153/326, 95% CI 41.4%-52.5%), 99.7% (325/326, 95% CI 98.3%-99.9%), and 10.7 (SD 5.9) minutes, respectively. In the questionnaire survey, 98.5% (321/326) of the patients reported that the tutorial was easy to understand, 96.0% (313/326) found stool image capture easy, and 87.8% (286/326) reported reduced anxiety regarding BP. Furthermore, 90.5% (295/326) of the patients indicated that they would like to use the app again for future colonoscopies. Among medical staff, 92.5% (62/67) considered the viewer system necessary, 89.6% (60/67) found it easy to use, and 89.6% (60/67) reported a reduction in workload burden.

**Conclusions:**

AI-based stool state assessment using the app and the viewer during BP was feasible across diverse BP methods and clinical environments. Favorable BP outcomes and high usability among patients and medical staff support the potential use of this approach in real-world colonoscopy practice.

## Introduction

The latest cancer statistics report high mortality and morbidity rates for colorectal cancer (CRC) worldwide [1]. Colonoscopy aids in the detection and resection of precancerous lesions, thereby reducing CRC-related morbidity and mortality [2-8]. Colonoscopy quality indicators include the adenoma detection rate (ADR), cecal intubation rate, withdrawal time, and bowel preparation (BP) [9-11]. Notably, the quality of BP is crucial for the other indicators to be maintained appropriately [12-15]. For instance, BP is associated with an improvement in ADR and with a decreased risk of CRC mortality [16].

Multiple factors affect BP quality, including patient adherence to laxatives and dietary instructions [17-19]. Accordingly, enhancing patient education using booklets, visual aids, short messages, or smartphone apps has proven effective [20-25]. Additionally, evaluating the stool state during BP is important to achieve appropriate BP quality. In Japan, BP is commonly performed at home or in the hospital. At home, patients use visual scales to assess their stool; in hospitals, the assessment may be conducted by the medical staff, contingent on the facility’s protocols. In both methods, colonoscopy is initiated when the stool is clear and watery. However, evaluating the stool state during BP can be burdensome for patients and medical staff, and an accurate evaluation may sometimes be difficult. Some patients may also feel embarrassed about having their stool evaluated by the medical staff; likewise, the medical staff may experience psychological discomfort when assessing the patient’s stool. Therefore, we previously developed a smartphone app incorporating AI to evaluate stool state during BP to address these issues, and the effectiveness of this app was demonstrated in a single-center study [26]. While the app was effective in assessing BP at the hospital, its feasibility at home or under diverse laxatives and toilet environments remains unknown. Furthermore, we developed a viewer system that connects to the app and enables the medical staff to easily monitor the patient’s stool state during routine tasks without directly checking the app screen. This system is intended to improve workflow efficiency in clinical settings.

Accordingly, in this study, we aimed to assess the feasibility of performing colonoscopy of appropriate quality using our app and viewer system.

## Methods

### Study Design

This multicenter prospective study was conducted at 10 Japanese institutions, comprising 6 tertiary hospitals, 3 regional general hospitals, and 1 community-based clinic, between November 2022 and December 2023. Consecutive patients scheduled to undergo colonoscopy at participating institutions during the study period were screened for eligibility. Eligible patients were approached by the medical staff at each institution and invited to participate in the study.

### Ethical Considerations

This study was conducted in accordance with the principles outlined in the Declaration of Helsinki and the Japanese Ethical Guidelines for Clinical Studies Involving Human Subjects. The study protocol was approved by the Institutional Review Board of the National Cancer Center (2022‐023). Written informed consent was obtained from all participants prior to study enrollment. All data were deidentified before analysis to protect participant privacy and confidentiality. Participants did not receive any financial compensation for participation in this study.

### Patient Selection

Patients who met the following criteria were included in the study: patients (1) who underwent colonoscopy at participating institutions, (2) aged between 20 and 70 years, and (3) who owned smartphones with Android or iOS compatibility. Patients were excluded from the study if they met any of the following criteria: (1) had a known entire or subentire circumferential CRC, (2) previously underwent colorectal resection (excluding appendectomy), (3) had inflammatory bowel disease, (4) had colorectal disease with active bleeding, (5) had an Eastern Cooperative Oncology Group performance status of ≥2, or (6) had severe dementia.

### Instructions to Use the App and the Viewer

**Figure 1.**
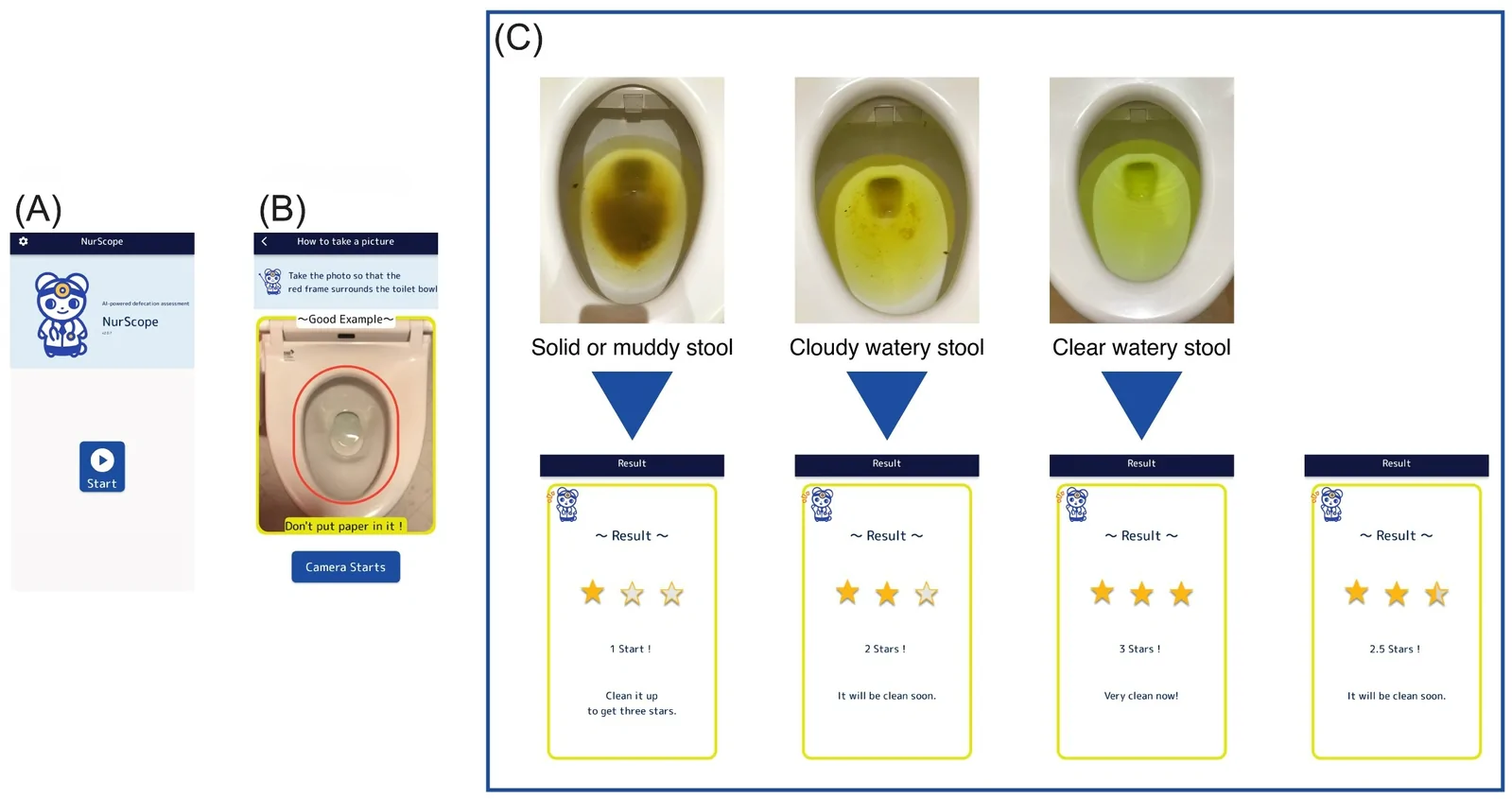
App screen display. (A) Start screen, (B) tutorial screen before taking pictures, and (C) screen displaying the result of stool image evaluation.

The screen displayed in the app is shown in Figure 1. The user was asked to start the app and tap the “Start” button. The user was presented with a screen explaining how to capture a picture. The user was cautioned to take a picture such that the red frame displayed on the screen aligned with the toilet bowl. Then, the user was asked to tap the button “Camera starts” to switch to the screen for taking pictures and touch the camera symbol to capture a picture. The captured stool images were graded on a 4-star scale: 1 star (solid or muddy stool), 2 stars (cloudy watery stool), 3 stars (clear watery stool), and 2.5 stars. Images evaluated as 1 or 2 stars indicated that the stools were insufficient to perform colonoscopy, whereas those evaluated as 3 stars indicated that the stools were sufficiently clear to perform colonoscopy. Images evaluated as 2.5 stars indicated clear watery stools excreted immediately after solid or muddy stools, or clear watery stools excreted despite a total defecation frequency of less than 5 times: this 2.5-star rating was included to ensure the reliability of the 3-star evaluation.

**Figure 2.**
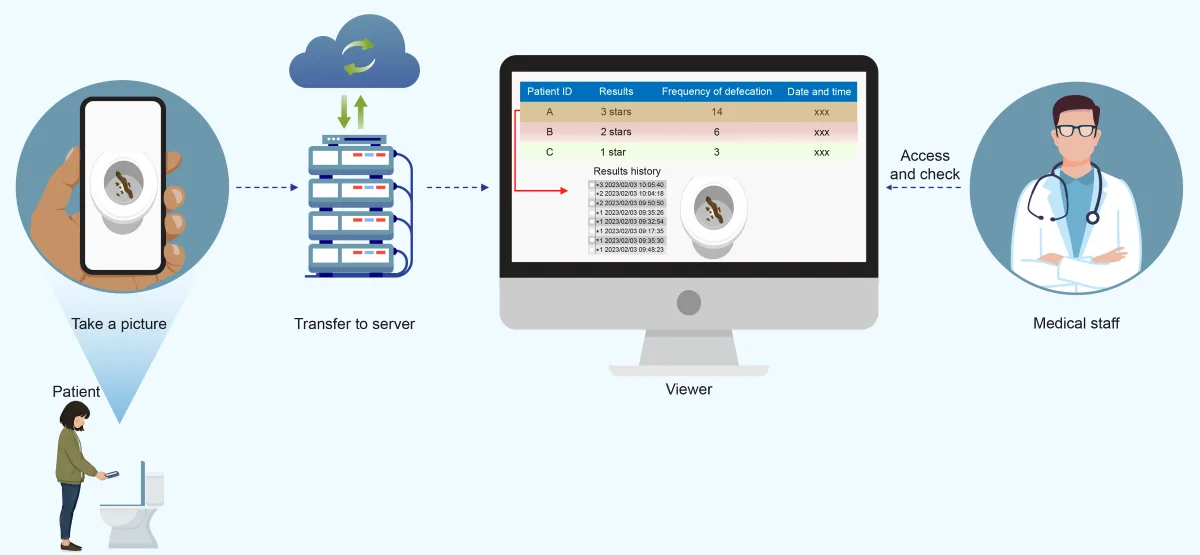
Overview diagram of the viewer system. Medical staff can check the number and timing of the patient’s defecations, as well as the app’s evaluation results, by entering the patient ID into the viewer.

The viewer system is shown in Figure 2. Medical staff accessed the viewer using a dedicated personal computer independent of the electronic medical record system at each institution. A unique patient ID was issued when a patient downloaded the app. The viewer screen showed the lists of patient ID, number of defecations, and evaluation of the latest stool state by the app. The medical staff selected the patient ID and checked the number of defecations, stool images, and the app’s evaluation. Further, the medical staff who participated in the study noted the patient ID in advance and checked the patient’s stool state and the app’s evaluation through the viewer during the BP, as necessary.

### Bowel Preparation

BP procedures followed the usual practices established at each institution. The method of laxative intake (same-day or split-dose method), the location where BP was performed, and the consumption of a low-fiber diet were determined by each institution. Patients received picosulfate, sennoside, magnesium oxide, or elobixibat the day before colonoscopy, as needed. On the day of colonoscopy, the patient was administered one of the following laxatives (Table S1 in Multimedia Appendix 1): polyethylene glycol with ascorbic acid (MOVIPREP; EA Pharma Co), magnesium citrate (MAGCOROL; Horii Pharmaceutical Ind), polyethylene glycol (NIFLEC; EA Pharma Co), oral sulfate solution (SULPREP; Fuji Pharma), or magnesium citrate plus sodium picosulfate (PICOPREP; Nippon Chemiphar Co). BP solutions were generally administered on the day of colonoscopy. However, when BP was performed over 2 days, including the day before and the day of colonoscopy, the regimen was determined according to the protocol of each participating institution. Prior to BP, patients downloaded the app to their smartphones and reviewed the instructions for use displayed within the app. Once BP was initiated, patients used the app to capture images of their stools every time they defecated. Colonoscopy was performed when the app presented 3 stars within 3 hours of administration. Contrastingly, when the app did not display 3 stars within 3 hours after BP initiation, additional procedures (such as additional laxative administration or enema) were conducted at the discretion of the doctor. If the app displayed 3 stars after additional procedures, a colonoscopy was subsequently performed. Colonoscopy was performed at the discretion of the doctor for patients who could not tolerate these procedures or who were not presented with 3 stars even after additional procedures.

### End Points

Considering that the quality of colonoscopy strongly depends on the degree of bowel cleansing, the Boston Bowel Preparation Scale (BBPS) was used to assess the quality of colonoscopy in this study [27]. The BBPS score was recorded by the endoscopists who performed the colonoscopy. Endoscopists were not blinded to study participation status or app evaluations. The primary end point was defined as the proportion of patients with a BBPS score of ≥6 among patients who successfully used the app. The secondary end points were the mean BBPS score; rate of excellent BBPS score (BBPS score ≥8); mean BBPS score of each segment (right colon, transverse colon, and left colon); ADR; cecal intubation rate; mean withdrawal time in negative colonoscopy; rate of fair, good, or excellent on the Aronchick scale [28]; mean time from laxative administration to colonoscopy initiation; proportion of patients with 3 stars, 2.5 stars, 2 stars, and 1 star for the app’s evaluation; proportion of cases with a BBPS score ≥6 when colonoscopy was performed for each evaluation in the app; diagnostic performance of the app for predicting adequate BP (BBPS score ≥6); mean BBPS score for each evaluation; and adverse events in colonoscopy. Additionally, we surveyed patients and medical staff who participated in this study to assess the usability and effectiveness of the app and viewer system using a questionnaire. These end points were also evaluated in patients who could successfully use the app, referring to patients who could launch the app by themselves, capture their stool images, and confirm the app’s evaluation. Successful app use was defined as obtaining at least 5 appropriate stool images during BP. If patients missed a stool image, they could self-report it using a dedicated function within the app. Cases were still considered successful even if some images were of poor quality, captured the stool only partially, or contained nonstool objects, provided that at least 5 appropriate stool images were ultimately obtained. We also calculated the proportion of patients who failed to use the app among all enrolled patients.

### Statistical Analysis

For the primary end point, the target value was defined as 95% or above according to the European guideline [10]. The threshold value for the proportion of patients with a BBPS score of ≥6 among patients who successfully used the app was 90%. Considering an expected value of 95%, a one-sided alpha of 2.5%, and a power of 90%, the number of patients required was calculated to be 316 by a method based on binomial distribution. The target number of patients was set to 340 to account for patients who could not use the app successfully. All statistical analyses were performed using SAS (version 9.4).

## Results

### Patient Characteristics

A total of 343 patients were enrolled in this study. After the exclusion of 17 patients, 326 eligible patients were included in the final analysis. The median patient age was 57 (IQR 48-63; range 26‐69) years, and 57.7% (n=188) were men (Table 1). Among the 326 patients, 99.4% (n=324) underwent same-day BP, and 68.4% (n=223) underwent BP at home. In addition, 8.6% (n=28) of the patients had a history of inadequate BP during a previous colonoscopy.

**Table 1.** Baseline characteristics of eligible patients (n=326).

Characteristics	Values
Age (y), median (IQR; range)	57 (48-63; 26‐69)
Sex, n (%)	
Male	188 (57.7)
Female	138 (42.3)
BMI (kg/m^2^), mean (SD)	23.1 (3.9)
Regular use of laxatives, n (%)	
Yes	17 (5.2)
No	309 (94.8)
Regular use of tricyclic antidepressant, n (%)	
Yes	1 (0.3)
No	325 (99.7)
History of abdominal (noncolorectal) surgery, n (%)	
Yes	58 (17.8)
No	268 (82.2)
Comorbidities, n (%)	
Diabetes mellitus	25 (7.7)
Liver cirrhosis	1 (0.3)
Cerebral stroke	7 (2.1)
Cerebral hemorrhage	1 (0.3)
Parkinson disease	0 (0)
History of inadequate bowel preparation, n (%)	
Yes	28 (8.6)
No	194 (59.5)
N/A due to no prior colonoscopy	96 (29.4)
Unknown	8 (2.5)
Indication of colonoscopy, n (%)	
Screening	148 (45.4)
Surveillance	114 (35.0)
Diagnostic	34 (10.4)
Symptomatic	26 (8.0)
Other	4 (1.2)
Methods of bowel preparation, n (%)	
Same-day bowel preparation	324 (99.4)
Split-dose bowel preparation	2 (0.6)
Location where the bowel preparation was performed, n (%)	
Patients’ home	223 (68.4)
Hospital	103 (31.6)
Taking a low-fiber diet, n (%)	
Yes	221 (67.8)
No	105 (32.2)
Laxative regimen, n (%)	
Polyethylene glycol with ascorbic acid	227 (69.6)
Magnesium citrate	48 (14.7)
Polyethylene glycol	28 (8.6)
Oral sulfate solution	21 (6.4)
Magnesium citrate plus sodium picosulfate	2 (0.7)

### Study Flow

**Figure 3.**
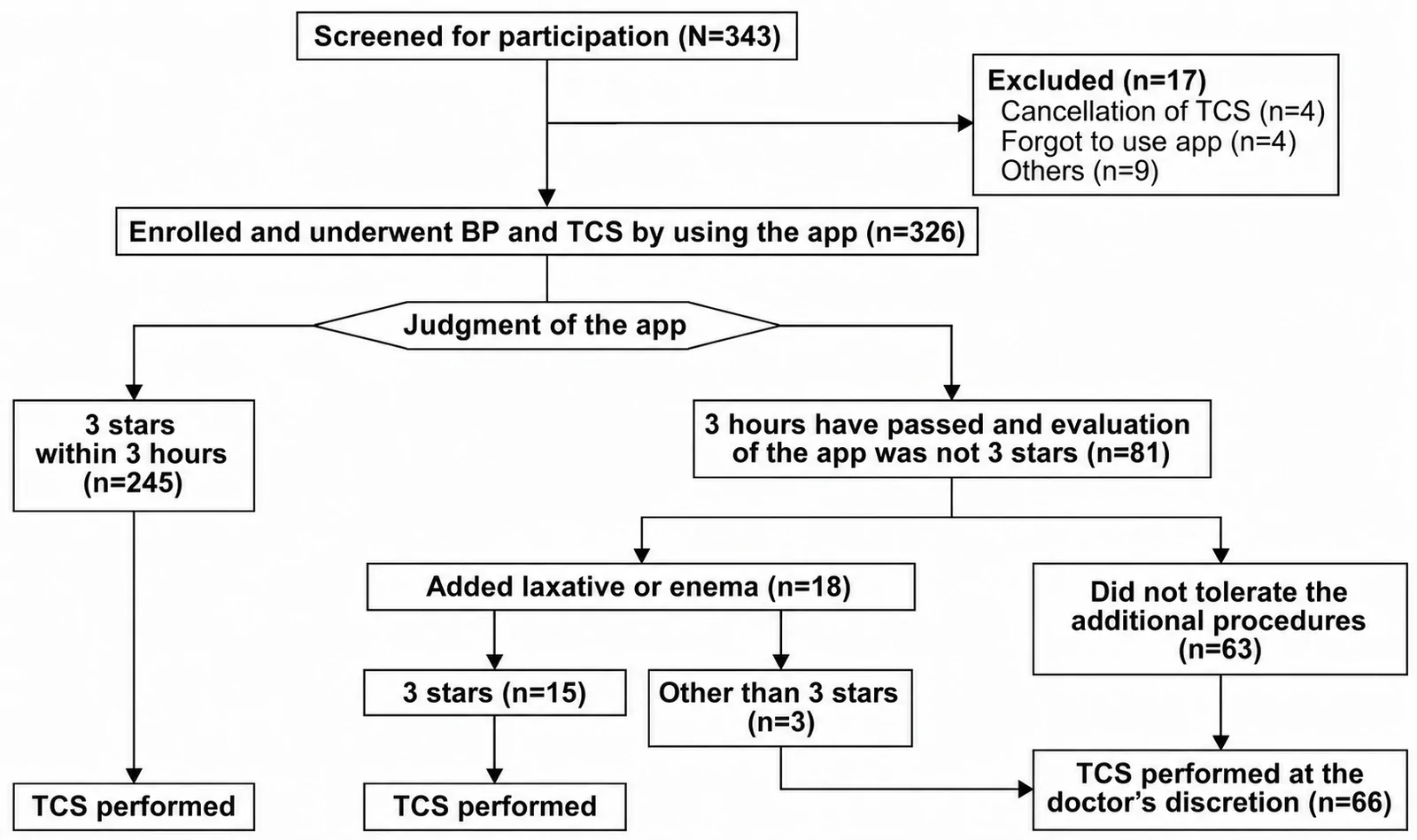
Status of the patients in the study flow from laxative administration to colonoscopy. BP: bowel preparation; TCS: total colonoscopy.

As shown in Figure 3, 326 patients successfully used the app and underwent colonoscopy following the app’s evaluation. Furthermore, 245 patients were conferred 3 stars within 3 hours of the start of laxative administration and subsequently underwent colonoscopy, whereas 81 patients did not receive 3 stars even 3 hours after laxative initiation. Of these, 63 patients who could not tolerate the additional procedure underwent colonoscopy at the discretion of the doctor, whereas 18 patients underwent additional procedures. Of these 18 patients, 15 were further evaluated with 3 stars, and a colonoscopy was performed. The remaining 3 patients who were not graded with 3 stars underwent colonoscopy at the discretion of the doctor.

### Outcome

Among the entire cohort, 99.1% (323/326, 95% CI 97.3%-99.8%) of the patients had a BBPS score of ≥6 (Table 2), indicating that the primary end point was met because the lower limit of the 95% CI exceeded the prespecified threshold of 90%. The mean BBPS score was 8.5 (SD 1.0), and the proportion of excellent BBPS score was 87.4% (285/326). The mean BBPS scores of the right, transverse, and left colons were 2.8 (SD 0.5), 2.9 (SD 0.3), and 2.9 (SD 0.4), respectively. The proportion of fair, good, or excellent Aronchick scale scores was 98.8% (322/326). The ADR, cecal intubation rate, and mean withdrawal time in negative colonoscopy were 46.9% (153/326, 95% CI 41.4%-52.5%), 99.7% (325/326, 95% CI 98.3%-99.9%), and 10.7 (SD 5.9) minutes, respectively. The proportions of patients with 3 stars, 2.5 stars, 2 stars, and 1 star for the app’s evaluation were 79.8% (260/326), 5.8% (19/326), 12.6% (41/326), and 1.8% (6/326), respectively. The proportion of cases with a BBPS score of ≥6 when colonoscopy was performed for each evaluation in the app was 100% (260/260), 100% (19/19), 95.1% (39/41), and 83.3% (5/6) for the 3 stars, 2.5 stars, 2 stars, and 1 star, respectively. The mean BBPS score for each evaluation outcome was 8.6 (SD 0.8), 7.9 (SD 1.2), 8.4 (SD 1.2), and 7.5 (SD 2.9) for 3 stars, 2.5 stars, 2 stars, and 1 star, respectively. The mean time from laxative administration to colonoscopy initiation was 305 (SD 89) minutes. The incidence rates of adverse events in colonoscopy, particularly bleeding, perforation, and ischemic colitis, were 0.6% (2/326), 0% (0/326), and 0% (0/326), respectively. The diagnostic performance of the app for predicting a BBPS score of ≥6 is summarized in Table S2 in Multimedia Appendix 1. The sensitivity, specificity, positive predictive value, and negative predictive value were 80.5%, 100%, 100%, and 4.5%, respectively.

**Table 2.** Outcomes of patients who used the app successfully (n=326).

Outcomes	Values
Primary end point	
Patients scoring BBPS^a^ score ≥6, n/N (%)	323/326 (99.1)
Secondary end points	
BBPS score, mean (SD)	8.5 (1.0)
Excellent bowel preparation (BBPS score ≥8) rate, n/N (%)	285/326 (87.4)
BBPS score of each segment, mean (SD)	
Right colon	2.8 (0.5)
Transverse colon	2.9 (0.3)
Left colon	2.9 (0.4)
Aronchick scale, n/N (%)	
1 (Excellent)	255/326 (78.2)
2 (Good)	53/326 (16.3)
3 (Fair)	14/326 (4.3)
4 (Poor)	3/326 (0.9)
5 (Inadequate)	1/326 (0.3)
Adenoma detection rate, n/N (%)	153/326 (46.9)
Cecal intubation rate, n/N (%)	325/326 (99.7)
Withdrawal time in disease-free patients (min), mean (SD)	10.7 (5.9)
Patients for app evaluation, n/N (%)	
3 stars	260/326 (79.8)
2.5 stars	19/326 (5.8)
2 stars	41/326 (12.6)
1 star	6/326 (1.8)
Rate of BBPS score ≥6 for each app evaluation, n/N (%)	
3 stars	260/260 (100)
2.5 stars	19/19 (100)
2 stars	39/41 (95.1)
1 star	5/6 (83.3)
BBPS score for each app evaluation, mean (SD)	
3 stars	8.6 (0.8)
2.5 stars	7.9 (1.2)
2 stars	8.4 (1.2)
1 star	7.5 (2.9)
Time from laxative administration to starting colonoscopy (min), mean (SD)	305 (89)
Adverse events, n/N (%)	
Bleeding	2/326 (0.6)
Perforation	0/326 (0)
Ischemic colitis	0/326 (0)

a BBPS: Boston Bowel Preparation Scale.

### Questionnaire Survey

Table 3 presents the results of the survey to assess the usability and effectiveness of the app and viewer system using a questionnaire administered to patients. Overall, 96.0% (313/326) of the patients answered that downloading the app was easy, 98.5% (321/326) reported that the description screen was easy to understand, and 96.0% (313/326) expressed that they could take pictures of their stool without any problems. Of the 15.6% (51/326) of patients who felt uncomfortable with the medical staff checking their defecation, 98.0% (50/51) of those answered that using the app would solve this problem. Furthermore, 37.7% (123/326) of the patients answered that they had no confidence in evaluating their own stool state, and 87.8% (108/123) of those patients answered that using the app would solve this problem. Ultimately, 90.5% (295/326) of the patients indicated a preference for using the app during their next colonoscopy.

**Table 3.** Questionnaire survey for patients (n=326).

Questionnaire results	Participants, n/N (%)
Question 1. Did you download the app without any problems?	
Yes	313/326 (96.0)
No	13/326 (4.0)
Question 2. Was the app tutorial screen easy to understand?	
Yes	321/326 (98.5)
No	5/326 (1.5)
Question 3. Was it easy to take stool images?	
Yes	313/326 (96.0)
No	13/326 (4.0)
Question 4. Was the evaluation of the app easy to understand?	
Yes	310/326 (95.1)
No	16/326 (4.9)
Question 5. Would you feel uncomfortable if your stool was checked by medical staff during the BP^a^?	
Yes	51/326 (15.6)
No	275/326 (84.4)
Did you feel that the app would improve your discomfort?^b^	
Yes	50/51 (98.0)
No	1/50 (2.0)
Question 6. Do you have any concerns about being able to evaluate your own defecation during the BP?	
Yes	123/326 (37.7)
No	203/326 (62.3)
Did you feel that your anxiety would be relieved by the app?^c^	
Yes	108/123 (87.8)
No	15/123 (12.2)
Question 7. Would you like to use the app for your next colonoscopy?	
Yes	295/326 (90.5)
No	31/326 (9.5)

a BP: bowel preparation.

b This question was administered to participants who answered "Yes" to question 5.

c This question was administered to participants who answered "Yes" to question 6.

Table 4 presents the results of the questionnaire survey administered to the medical staff. Overall, 83.6% (56/67) of the medical staff checked the patients’ stool state during daily work. Among them, 17.9% (10/56) answered that they felt a psychological burden, and 55.4% (31/56) answered that they felt a physical burden in checking patients’ stool state in their daily work. Notably, 89.6% (60/67) of the medical staff indicated that the implementation of the app would alleviate these burdens. For the viewer system, 89.6% (60/67) of the medical staff reported that it was easy to use, while 92.5% (62/67) indicated that it was necessary for BP.

**Table 4.** Questionnaire survey for medical staff (n=67).

Questionnaire results	Participants, n/N (%)
Question 1. Do you check the patients’ stool state during BP^a^ in your daily work?	
Yes	56/67 (83.6)
No	11/67 (16.4)
Do you have a psychological burden to check patients’ stool state?^b^	
Yes	10/56 (17.9)
No	46/56 (82.1)
Do you have a workload to check patients’ stool state?^b^	
Yes	31/56 (55.4)
No	25/56 (44.6)
Question 2. Did you feel that using the app during BP would reduce the workload of checking patients’ stools?	
Yes	60/67 (89.6)
No	7/67 (10.4)
Question 3. How was the operability of the viewer screen displaying?	
Easy	60/67 (89.6)
Difficult	7/67 (10.4)
Question 4. Did you feel that the viewer screen was necessary?	
Yes	62/67 (92.5)
No	5/67 (7.5)

a BP: bowel preparation.

b This question was administered to participants who answered "Yes" to question 1.

## Discussion

This multicenter prospective study successfully evaluated the performance of the app across various types of laxatives, toilet bowls, and diverse patient populations. Among 326 patients who successfully used the app, 99.1% (n=323) patients achieved a BBPS score ≥6, which exceeded the set expected value. Additionally, several key quality indicators, including an ADR of 46.9% (n=153) and a cecal intubation rate of 99.7% (n=325), exceeded the target values recommended by American and European guidelines [9,10]. These results demonstrated the feasibility of app-guided BP management while maintaining high-quality colonoscopy indicators across multiple institutions and environments.

Notably, this study demonstrated the effective use of our app across a wide range of clinical settings. Specifically, we validated our app under diverse BP protocols and toilet environments and demonstrated that it could be applied to BP management in various clinical contexts. The following 2 main findings of this study support the versatility of the app, which is critical for its implementation in real-world clinical practice.

First, we evaluated the app’s assessment of BP using a variety of laxatives. In Japan, 6 major types of laxatives are commonly used for BP [29], of which 5 were used in this study. Additionally, the timing of administration, use of a low-fiber diet, and administration of laxatives on the day before colonoscopy were determined according to the protocols of each institution. Regardless of the type of laxative used and a wide variety of BP methods, a stool state evaluation rated as 3 stars using the app indicated that colonoscopy could be performed with a BBPS score ≥6 in 100% (260/260) of cases. Moreover, the mean BBPS score of patients rated as 3 stars was ≥8 (SD 0.8; excellent BBPS score). Even in real-world clinical settings, which exhibit variability in the types of laxatives used and BP methods, the 3-star rating by the app was shown to be highly correlated with an excellent BBPS score. These findings suggest that the app can assist BP across a broad range of institutional practices (eg, clinics and general hospitals) and diverse patient needs (eg, preference and tolerability for specific laxatives and low-fiber diets) while maintaining an adequate quality of colonoscopy. Although the app evaluations were associated with BP quality, the app was not designed to directly predict BBPS score. Instead, the app was intended to evaluate stool state to verify whether BP was sufficient to proceed with colonoscopy. In the present study, 95.1% (39/41) of the patients with a 2-star evaluation and 83.3% (5/6) of those with a 1-star evaluation still achieved a BBPS score ≥6. Consistent with these findings, the app showed a high positive predictive value (100%) but a low negative predictive value (4.5%) for predicting a BBPS score of ≥6 above. These results suggest that while a 3-star evaluation reliably identified patients with adequate BP (BBPS score ≥6), lower star evaluations did not necessarily indicate inadequate BP (BBPS score <6). Although the number of patients with a 1-star evaluation was small, these findings suggest that a substantial proportion of patients with lower star evaluations ultimately achieved adequate BP. This may reflect limitations of the star-based evaluation, as well as the imperfect relationship between stool state immediately before colonoscopy and overall bowel cleanliness assessed by the BBPS. Nevertheless, further refinement of the stool-state assessment algorithm may improve its ability to distinguish between different stool states during BP. In the present study, some patients with 1-star or 2-star evaluations still achieved a BBPS score of ≥6, suggesting that additional preparation may have been unnecessary in a subset of cases. Therefore, there remains room for improvement in the diagnostic discrimination of the app, particularly in reducing potential additional preparation (eg, additional laxative intake or enema). To further clarify the diagnostic characteristics of the app, sensitivity, specificity, positive predictive value, and negative predictive value for predicting a BBPS score of ≥6 are summarized in Table S2 in Multimedia Appendix 1.

Second, the study included both hospital-based and home-based BP settings, reflecting diverse real-world environments with potential variability in toilet conditions. Unlike our previous study [26], this study also included patients who underwent BP at home. Evaluations for BP at hospitals were conducted using toilet bowls available at each of the 10 participating institutions. Meanwhile, evaluations for BP at home were performed using toilets installed in each patient’s home. Thus, the app assessed a wide variety of toilet bowls. In both settings, when the evaluation of the app was 3 stars, colonoscopy was performed with a BBPS score of ≥6 in 100% (260/260) of the cases. Furthermore, the mean BBPS score for patients rated as 3 stars was ≥8 (SD 0.8) in both settings, indicating that an excellent BBPS score was achieved. Therefore, even across a wide variety of toilet environments with differences in shape, color tone, and lighting conditions, the app was able to evaluate stool state consistency; when the stool was rated as 3 stars, colonoscopy could be performed with an adequate BBPS score. These findings suggest that the app may be applicable across diverse clinical and home-based environments and support its potential feasibility in real-world clinical practice.

Using a similar approach to this study, 2 recent reports have demonstrated the effectiveness of AI-based smartphone apps in improving BP quality [30,31]. In previous studies, the developed app had a binary classification function directly determining whether the bowel cleansing quality was adequate based on the stool state. Consequently, the mean BBPS score achieved with those apps was 7.2 (SD 1.4) and 6.7 (SD 1.3), respectively. In contrast, our app evaluated each defecation individually during the BP process, considering the number of defecations. The app was used to assess stool state throughout the BP process, followed by colonoscopy. In our study, a mean BBPS score of 8.5 (SD 1.0) was observed, which was higher than the values reported in previous studies. These findings suggest that it is difficult to accurately predict the quality of BP solely based on a single stool state and that evaluating all defecations may support more appropriate BP management throughout the BP process.

The observed rate of the BBPS score of ≥6 in this study was notably high (99.1%, 323/326), exceeding both the minimum (≥90%) and target (≥95%) standards recommended by the European guideline [9,10]. Several factors may have contributed to this finding. First, patients aged 70 years or older were excluded from this study. Because advanced age is a known risk factor for inadequate BP, this exclusion may have contributed to the favorable BBPS outcomes. However, this exclusion criterion was not intended to select patients with better BP quality. Instead, this decision was based on the low smartphone ownership rates among older individuals in Japan at the time of study protocol development (38.3% among individuals in their 70s and 11.0% among those in their 80s), as well as our previous single-center experience showing that all patients who were unable to appropriately operate the app were aged 70 years or older [26,32]. Therefore, older patients were excluded because they were considered more likely to have difficulty appropriately using the app in this study. In addition, the use of the app itself and the awareness that stool images and app evaluations could be reviewed by medical staff through the viewer system may have increased patient awareness and adherence to BP instructions (ie, observation effect).

Although the mechanisms underlying the potential benefits of the app were not directly evaluated in this study, several possible explanations and future perspectives may be considered. Several mechanisms may explain how the app could support BP management. First, the viewer system enabled real-time monitoring of stool state during BP, allowing medical staff to review temporal changes and provide timely instructions when needed. Second, the use of the app itself may have promoted behavioral reinforcement and improved adherence to BP instructions. In addition, future expansion of app functions, such as automated reminders for examination schedules and dietary instructions, may further support behavioral modification during BP. Third, the app may help reduce uncertainty in BP assessment. In Japan, stool state is commonly evaluated during BP; however, assessment methods may vary across institutions and between patients and medical staff. By providing standardized stool state assessment and visualization of temporal changes in stool state, the app may help reduce variability and uncertainty in BP management.

Although our app evaluates the stool state over time during BP, the responsibility for deciding whether to proceed with colonoscopy based on the app’s evaluations or to implement additional procedures if the BP is insufficient lies with the medical staff. Therefore, a viewer system was introduced in this study to enable an efficient review of temporal changes in stool state and the corresponding evaluations during BP. In the questionnaire survey, most medical staff reported no issues with the usability of the viewer and responded that the viewer was necessary during BP. Particularly, the reasons cited for the necessity of the viewer were that its use enabled real-time monitoring of BP, allowing timely instructions for patients during the BP process, and that it had the potential to reduce patient waiting times before colonoscopy. Thus, by enabling a standardized assessment of the stool state and real-time monitoring, the combined use of the app and viewer system introduces a new framework for quality assurance in BP.

Finally, the usability of our app was evaluated using questionnaires administered to patients and medical staff. Most patients were able to use the app without any issues, and discomfort with having their stool state assessed by medical staff, as well as anxiety about evaluating their own stool state, was alleviated using the app. The observed reduction in patient anxiety during BP suggests that the app may also help lower the psychological barriers to undergoing colonoscopy. Furthermore, answers from medical staff suggested that using the app might help reduce their physical and psychological burdens. This reduction implies that the app can support a more efficient clinical workflow. In health care systems experiencing workforce shortages, especially in aging populations, tools that facilitate task shifting and reduce manual workloads are essential. In this regard, our app may provide a practical solution for supporting efficient BP management without compromising the quality of care.

Nevertheless, this study has some limitations. First, the study was not designed as a randomized controlled trial and therefore lacks direct comparison with BP without the use of the app. Future randomized controlled trials comparing outcomes with and without the app are essential to validate the clinical usage of the app more robustly. Second, we did not specifically assess the usability of the app in patients aged 70 years or older. This subset of patients was excluded from the study because smartphone ownership rates among older individuals in Japan were relatively low at the time of study initiation, and many older patients were unfamiliar with smartphone app operation. In addition, in our previous single-center study, all patients who were unable to appropriately operate the app were aged 70 years or older. However, smartphone ownership among older individuals in Japan has increased substantially in recent years. According to national data from 2024, smartphone ownership rates have risen to 67.5% among individuals in their 70s and 30.7% among those in their 80s [33]. Therefore, with further improvements in user interface design and usability support, the applicability of the app to older patients and populations with lower digital literacy may expand in the future. Third, this study was conducted exclusively in Japan. Therefore, the generalizability of the results to other countries remains unclear. Furthermore, because BP protocols and toilet environments differ across countries and regions, further validation studies are needed to confirm whether the app can achieve similar performance outside Japan. Fourth, endoscopists who assigned BBPS were not blinded to study participation status or app evaluations. Therefore, the possibility of observer bias in BBPS assessment cannot be excluded because the primary end point relied on endoscopist-assigned BBPS. Although all participating endoscopists received standardized instruction on BBPS assessment, including washing and suctioning of residues before scoring, such training cannot completely eliminate the possibility of assessment bias in a nonblinded study. Fifth, the primary end point analysis included only patients who successfully used the app. Therefore, the observed results may overestimate the feasibility of app-guided BP management in real-world settings.

In conclusion, the proportion of patients with a BBPS score of ≥6 for colonoscopy using the app exceeded the expected value in addition to the threshold value. In this multicenter prospective study, AI-based stool state assessment using the app and viewer during BP was feasible across diverse BP methods and clinical environments. Favorable BP outcomes and high usability among patients and medical staff support the potential usage of this approach in real-world colonoscopy practice.

## Supplementary material

10.2196/91274 Multimedia Appendix 1 Laxative administration protocols and diagnostic performance of the app.

## Abbreviations

ADR: adenoma detection rate
BBPS: Boston Bowel Preparation Scale
BP: bowel preparation
CRC: colorectal cancer
