# Supplementary material for: Feasibility of a Stool State Check App Using AI During Bowel Preparation Before Colonoscopy: Multicenter Prospective Study (SCAN Study)
Source: JMIR Mhealth Uhealth. 2026 Sep 1;14:e91274. doi: 10.2196/91274 (PMC13533039; doi:10.2196/91274)
Supplement: Supplementary file 1 [file mhealth-v14-e91274-s001.docx]

**Supplementary Table 1. Standard dosing and administration protocols for each laxative**

| **Generic name** | **Protocol** |
| --- | --- |
| Polyethylene glycol with ascorbic acid | The solution should be administered orally at a rate of approximately 1 L per hour, followed by approximately 0.5 L of water or tea. |
| Magnesium citrate | A total of 1,800 mL of the solution should be administered orally in 200-mL portions over approximately 1 hour, starting at least 4 hours before the scheduled examination time. |
| Polyethylene glycol | Approximately 2–4 L of the solution should be administered orally at a rate of approximately 1 L per hour. |
| Oral sulfate solution | 480 mL of the solution should be administered orally over 30 minutes, followed by approximately 1 L of water or tea over 1 hour. |
| Magnesium citrate plus sodium picosulfate | One sachet should be dissolved in approximately 150 mL of water and administered twice. After the first dose, at least five servings of 250 mL of clear fluids should be consumed over several hours. After the second dose, at least three servings of 250 mL of clear fluids should be consumed before 2 hours before the examination. |

**Supplementary Table 2. Diagnostic performance of the app for predicting adequate bowel preparation (BBPS ≥ 6)**

| **Parameter** | **Definition** | **Value (%)** |
| --- | --- | --- |
| Sensitivity (%), n | TP / (TP+FN) | 80.5 (262/326) |
| Specificity (%), n | TN / (TN+FP) | 100 (3/3) |
| Positive predict value (%), n | TP / (TP+FP) | 100 (262/262) |
| Negative predictive value (%), n | TN / (TN+FN) | 4.5 (3/67) |

BBPS: Boston Bowel Preparation Scale, TP: True positive, TN: True negative, FP: False positive, FN: False negative.

Adequate bowel preparation was defined as BBPS ≥ 6.

Three-star app evaluation is considered predictive of adequate bowel preparation.

TP indicates 3-star evaluation with BBPS ≥ 6; FN, non–3-star evaluation with BBPS ≥ 6; FP, 3-star evaluation with BBPS < 6; and TN, non–3-star evaluation with BBPS < 6.
